# Associations Between Serum Fatty Acids and Immunological Markers in Children Developing Islet Autoimmunity—The TRIGR Nested Case–Control Study

**DOI:** 10.3389/fimmu.2022.858875

**Published:** 2022-05-25

**Authors:** Sari Niinistö, Maija E. Miettinen, David Cuthbertson, Jarno Honkanen, Leena Hakola, Reija Autio, Iris Erlund, Petra Arohonka, Arja Vuorela, Taina Härkönen, Heikki Hyöty, Jeffrey P. Krischer, Outi Vaarala, Mikael Knip, Suvi M. Virtanen, TRIGR Investigators

**Affiliations:** ^1^ Department of Public Health and Welfare, Finnish Institute for Health and Welfare, Helsinki, Finland; ^2^ Health Informatics Institute, Morsani College of Medicine, University of South Florida, Tampa, FL, United States; ^3^ Research Program for Clinical and Molecular Metabolism, Faculty of Medicine, University of Helsinki, Helsinki, Finland; ^4^ Unit of Health Sciences, Faculty of Social Sciences, Tampere University, Tampere, Finland; ^5^ Tampere University Hospital, Research, Development and Innovation Center, Tampere, Finland; ^6^ Department of Government Services, Finnish Institute for Health and Welfare, Helsinki, Finland; ^7^ Faculty of Medicine and Health Technology, Tampere University, Tampere, Finland; ^8^ Fimlab Laboratories, Pirkanmaa Hospital District, Tampere, Finland; ^9^ Pediatric Research Center, Children’s Hospital, University of Helsinki and Helsinki University Hospital, Helsinki, Finland; ^10^ Research Program for Clinical and Molecular Metabolism, University of Helsinki, Helsinki, Finland; ^11^ Center for Child Health Research, Tampere University and Tampere University Hospital, Tampere, Finland

**Keywords:** fatty acids, immunological markers, cytokines, chemokines, growth factors, islet autoimmunity, children, type 1 diabetes

## Abstract

**Aims:**

Altered immune functions as well as fatty acid intake and status have been associated with the development of type 1 diabetes. We aimed to study the relationship between fatty acids and immunological markers in young children with increased genetic risk for type 1 diabetes in order to define putative mechanisms related to development of islet autoimmunity.

**Methods:**

Serum samples for fatty acid and immunological marker measurements were obtained in the Trial to Reduce IDDM in the Genetically at Risk (TRIGR) ancillary study (Divia) from children born between 2002 and 2007 in 15 countries. Case children (*n* = 95) were defined as having repeated positivity for at least two out of four diabetes-associated autoantibodies. For each case child, control children were selected matched for country and date of birth (*n* = 173). Serum fatty acids and immunological markers were measured from cord serum and at the age of 6 and 12 months. Spearman correlation coefficients were calculated between fatty acids and immunological markers.

**Results:**

Correlations between circulating fatty acids and immunological markers were different in case children who developed islet autoimmunity than in control children already at birth continuing across the first year of life. In case children, saturated fatty acids (SFAs) showed stronger correlations with immunological markers, while in controls, polyunsaturated fatty acids (PUFAs) showed stronger correlations.

**Conclusions:**

In cases, SFAs were associated with several immunological markers (CXCL10, IL-6, IL-9, IL-17, and CM-CSF) previously linked to the type 1 diabetes disease process. Findings indicate that fatty acids could have immunomodulatory potential in the early phase of the disease development, although causality between fatty acids and the immunological pathways remains to be explored.

**Trial registry number:**

NCT00179777

## Introduction

Type 1 diabetes is an autoimmune disease in which the immune system erroneously attacks and destroys the insulin-producing pancreatic beta cells (1). Circulating immunological markers and fatty acids have been linked to the development of type 1 diabetes (2–11). Immunological markers include cytokines, chemokines, and growth factors, which are soluble messenger molecules produced by various cell types. They are crucial regulators of immune functions inducing dynamic changes in the phenotype, functioning, and migration characteristics of immune cells. While being elementary for the regulation of immune cells, these factors also regulate various functions of the tissues under inflammatory signals (12, 13). Fatty acids also play a role in several physiological reactions, and their circulating levels are affected by diet and endogenous synthesis and metabolism, which again can be affected by genes, body weight, and health status, for example (14).

Alterations in immune function, such as overactivation of Th1 and Th17 immunity as well as abnormal cytokine production, have been related to the etiology of type 1 diabetes, although the detailed pathogenesis is poorly known (15, 16). Increased levels of IFN-γ, IL-17, IL-9, IL-6, IL-1β, CCL2 (MCP1), CCL4, and CXCL10 (IP-10) (3–6, 15, 17), as well as activation and phenotypic plasticity of Th17 cells have been associated with the development of islet autoimmunity and progression from the autoantibody-positive state towards clinical type 1 diabetes (18, 19). The role of Th17 plasticity in the development of type 1 diabetes was further implicated by the observation that TGF-β induced secretion of IL-9 in Th17 cells in children with recent-onset type 1 diabetes (7, 20, 21). Children with newly diagnosed type 1 diabetes had elevated levels of granulocyte macrophage-colony stimulating factor (GM-CSF), IL-2, IL-7, IL-8, IL-1β, IL-17F, IL-21, IL-23, IL-10, and IL-27 compared to control children (6, 8). Also, *in vitro* studies have indicated that certain cytokines are linked to type 1 diabetes. IL-17 alone or in combination with IL-1β and IFN-γ has been demonstrated to be detrimental to human islets and beta cells (15, 19, 22). Some evidence indicates that cytokine production is altered already before the appearance of islet autoimmunity: type 1 IFN-mediated innate immune system has been shown to be activated (23) and IL-32 upregulated (24). Furthermore, high maternal levels of circulating inflammatory cytokines (IFN-γ, IL-1β, CCL2, and CCL4) during pregnancy have been associated with increased risk of type 1 diabetes in the offspring (4, 17). In addition to immunological alterations, also early metabolic dysregulation of lipids and amino acids in cord blood or in infancy have been linked to type 1 diabetes (25–27). Furthermore, several abnormalities including alterations in lipid metabolism and inflammatory events were seen in children already 1 year before seroconversion (11).

Evidence from prospective cohort studies suggests that fatty acids contribute to the risk of islet autoimmunity, although the findings are partly inconsistent (9, 10, 28–31). High levels of long-chain n-3 polyunsaturated fatty acids (PUFAs) in serum or in the erythrocyte membrane might protect from islet autoimmunity (9, 10, 31), especially at an early age (10, 31). On the other hand, in the TRIGR study, we observed that high serum proportions of saturated fatty acids (SFAs) (15:0, i17:0, ai17:0, 18:0) and conjugated linoleic acid (CLA, 18:2n-7) were associated with increased risk of islet autoimmunity (30). Higher levels of some SFAs and MUFAs were also associated with an enhanced risk of islet autoimmunity in two other large-scale birth cohort studies (DIPP and TEDDY) (29, 31).

Fatty acids have been observed to affect both innate and adaptive immunity in *in vitro* studies and in animal models. Fatty acids may modulate immune system, e.g., by activating or downregulating inflammatory processes such as cytokine secretion (32). In general, n-3 PUFAs are considered as anti-inflammatory, and SFAs are considered as pro-inflammatory, based on *in vitro* and animal studies (32). In human adults, supplementation with long-chain n-3 PUFAs decreased levels of TNF-α, IL-6, and CRP according to a meta-analysis (33). To our knowledge, there is only one intervention study observing the effects of fatty acids on the participating children’s immunological markers: supplementation with DHA during pregnancy or infancy had no effect on the production of inflammatory cytokines such as IL-1β, TNF-α, or IL-12p40 in the offspring (34). As far as we are aware, no information exists on the associations between fatty acids and immunological markers during infancy, and whether these associations are different in children who develop islet autoimmunity and those who do not.

Our aim was to study the associations between serum fatty acids and immunological markers at birth and at the age of 6 and 12 months, and to study whether these associations differed between case and control children in order to define putative mechanisms related to development of islet autoimmunity. We hypothesized that n-3 PUFAs are associated with higher levels of anti-inflammatory immunological markers during infancy, while SFAs are related to pro-inflammatory immunological markers.

## Research Design and Methods

### Study Population

The current case–control study (Divia) is nested within the Trial to Reduce IDDM in the Genetically at Risk (TRIGR) cohort. TRIGR is an international double-blind randomized clinical trial of 2,159 infants with HLA-conferred disease susceptibility and a positive family history for T1D recruited between 2002 and 2007 in 15 countries followed until the youngest child turned 10 years of age (35). The inclusion criteria of the TRIGR study included both certain HLA-conferred genotypes known to increase the risk of type 1 diabetes and a first-degree relative affected by type 1 diabetes (35, 36). TRIGR tested whether weaning to an extensively hydrolyzed casein-based infant formula compared to a regular cow’s milk-based one during the first 6–8 months of life protected from type 1 diabetes. Blood samples were collected at 3- to 12-month intervals. In the Divia ancillary study, associations between the immune system, diet, and virus infections in the development of type 1 diabetes are studied. The definition of cases was positivity for at least two autoantibodies out of ICA, IAA, IA-2A, and GADA. For each case child (*n* = 244), two control children (*n* = 488) were randomly selected from children who did not fulfill the case criteria. Control children were matched for date of birth (± 1 year) and country. From this original nested case–control setting, those children who had unthawed serum samples available at birth and at the age of 6 or 12 months were included in the current study (case children, *n* = 95; control children, *n* = 173). Autoantibodies were quantified with the use of specific radiobinding assays in the Scientific Laboratory, Children’s Hospital, University of Helsinki, Helsinki, Finland (36). Written informed consent was collected from all families, signed by the legal guardian of the child. The study was approved by the ethics committees of all participating centers (see **Supplementary Data Sheet 2**). The study was conducted according to the standards of the Declaration of Helsinki.

### HLA Genotyping

HLA genotyping for the selected DQB1 and DQA1 alleles was performed using sequence-specific oligonucleotide hybridization with the following genotypes regarded as eligible: (1) HLA DQB1*02/DQB1*03:02 (high risk); (2) HLA DQB1*03:02/x (*x* not DQB1*02, DQB1*03:01, or DQB1*06:02) (moderate risk); and (3) HLA DQA1*05-DQB1*02/y (*y* not DQA1*02:01-DQB1*02, DQB1*03:01, DQB1*06:02, or DQB1*06:03) or HLA DQA1*03-DQB1*02/y (*y* not DQA1*02:01-DQB1*02, DQB1*03:01, DQB1*06:02, or DQB1*06:03) (mild risk) (35).

### Serum Fatty Acid Analysis

Serum fatty acids were analyzed at the Department of Government Services at the Finnish Institute for Health and Welfare, Helsinki, Finland. Fatty acids were determined by gas chromatography using an Omegawax 320 column (Supelco, Sigma-Aldrich Group) from serum samples collected at the age of 0, 6, and 12 months between the years 2002 and 2008. First, lipids were extracted from serum by liquid–liquid extraction (methanol/dichloromethane/sodium chloride). After that, fatty acids were methylated using acid-catalyzed procedures after which solid-phase extraction was performed to remove cholesterol esters. The samples were stored frozen in −70°C until analyzed. The samples were analyzed according to a predetermined list, with the matched cases and controls sorted together to minimize variation in results.

### Analyses of Serum Immunological Markers

**Figure 1:**
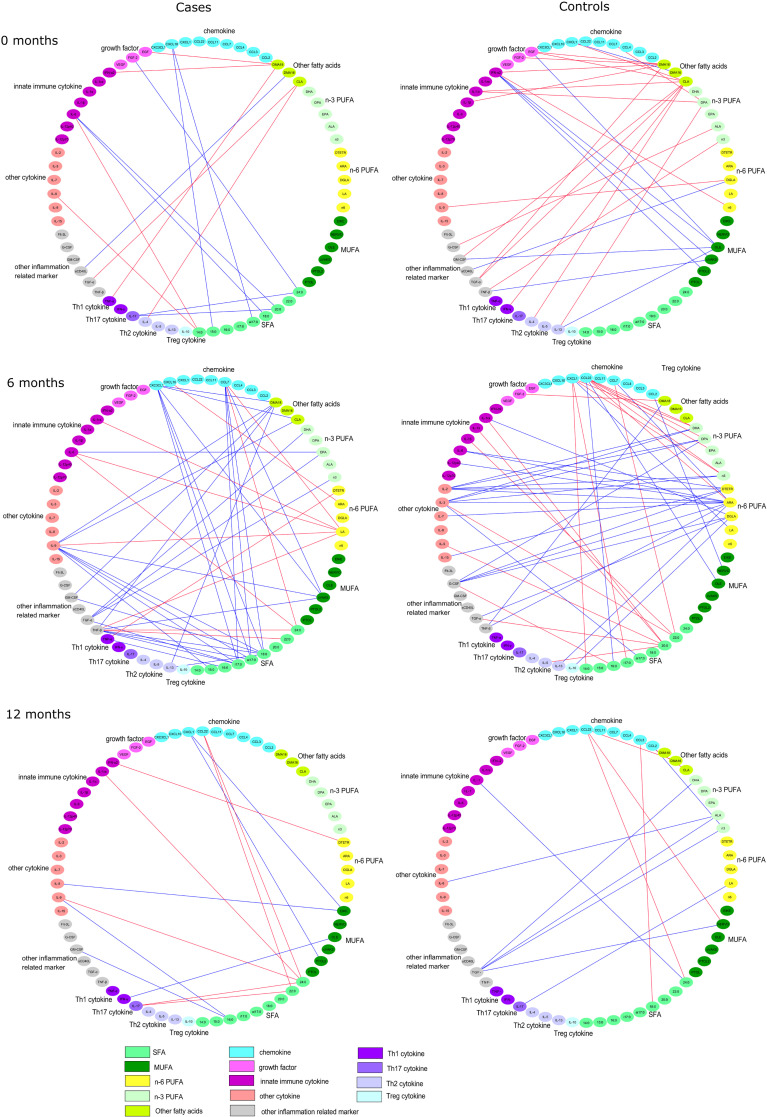
Spearman correlation coefficients between serum fatty acids and immunological markers separately in case and in control children in cord serum, and at the ages of 6 and 12 months. All the direct correlations are marked with red edges, while the inverse correlations are marked with blue edges with a cutoff of *p* < 0.01.

The serum concentrations of cytokines, chemokines, and growth factors were analyzed from unthawed serum samples with Luminex technology using the 38-plexed Milliplex MAP Kit (cat.no. HCYTMAG-60K-PX38) according to the manufacturer’s recommendations (Merck-Millipore Corp., Billerica, MA, USA). Luminex analyses were performed with single reactions using undiluted serum samples. Quantification of the markers was performed with Bio-plex 200 Luminex instrument and Bio-Plex Manager software (Bio-Rad, Sweden). The concentration of each marker was determined from an 8-point standard curve using five-parameter logistic regression. Minimum detectable concentration (MinDC) was determined for each marker separately using the lowest concentration on the standard curve linear phase [MinDC=c(low)+2SD]. The samples below MinDC were given a value of 50% of the MinDC. We categorized the cytokines into six groups: Th1, Th2, Th17, and Treg cytokines; chemokines; growth factors; innate immune cytokines; other cytokines; and other inflammation-related markers (**Figure 1**).

### Statistical Analyses

Spearman correlation coefficients between fatty acids and immunological markers were calculated separately among the case and control children. Serum fatty acid proportions were transformed using a centered log-ratio transformation. The analyses were performed using SAS v9.4. Since there were a high number of statistically significant correlations between immunological markers and fatty acids, we decided to use 0.01 as the significance level (**Figure 1**). The Spearman correlations were illustrated with the Cytoscape (Version 3.7.2). Only the correlations with a *p*-value <0.01 for either cases or controls were selected for the correlation comparison analysis (**Tables 2**–**4**). The differences within these Spearman correlations between cases and controls were compared using Fisher’s *r* to *z*-transformation for Spearman correlation and *z*-test (*p* < 0.05), and *p*-values were further adjusted with the Benjamini–Hochberg method (37, 38).

## Results

The median (interquartile range) age at positivity for at least two antibodies was 4.0 (1.9–6.0) years among the 95 case children. One child tested positive for at least two antibodies already at the age of 6 months and 26 children at the age of 12 months. The most common autoantibody combinations among the case children at the time of case selection were ICA and GADA (*n* = 26); ICA and IAA (*n* = 19); ICA, IAA, and GADA (*n* = 15); ICA, IAA, GADA, and IA-2A (*n* = 15); IAA and GADA (*n* = 10); and ICA, GADA and IA-2A (*n* = 6). One child had IAA, GADA, and IA-2A; one had ICA and IA-2A; one had ICA, IAA, and IA-2A; and one child had ICA only but presented with type 1 diabetes. Characteristics of the participants are presented in **Table 1**. Distributions of serum fatty acid proportions are presented in **Supplementary Table 1** and those of immunological markers in **Supplementary Table 2**.

**Table 1:** Characteristics of children with islet autoimmunity and control children in the TRIGR Divia Study.

	Case children, *n* = 95 *n* (%)	Control children, *n* = 173 *n* (%)
Sex		
Male	54 (56.8%)	92 (53.2%)
Female	41 (43.2%)	81 (46.8%)
HLA risk^*^		
High risk	38 (40.0%)	42 (24.3%)
Moderate risk	37 (38.9%)	67 (38.7%)
Mild risk	20 (21.1%)	64 (37.0%)
Family member with type 1 diabetes		
Mother alone or with father or sibling	32 (33.7%)	85 (49.1%)
Father or/and full sibling	63 (66.3%)	88 (50.9%)
Treatment group in TRIGR study		
Casein hydrolysate	56 (58.9%)	79 (45.7%)
Control formula	39 (41.1%)	94 (54.3%)
Country or region		
Northern Europe (Finland, Sweden)	25 (26.3%)	46 (26.6%)
Central Europe I (Czech Republic, Estonia, Hungary, Poland)	25 (26.3%)	49 (28.3%)
Central Europe II (Germany, Luxembourg, Netherlands, Switzerland)	9 (9.5%)	17 (9.8%)
Southern Europe (Italy, Spain)	0 (0%)	0 (0%)
Canada	13 (13.7%)	20 (11.6%)
United States	17 (17.9%)	30 (17.3%)
Australia	6 (6.3%)	11 (6.4%)
Any breastfeeding		
0–5.9 months	32 (33.7%)	54 (31.2%)
≥6 months	63 (66.3%)	119 (68.8%)

^*^High risk: HLA-DQB1*0302/DQB1*02 Moderate risk: HLA-DQB1*0302/x (x not DQB1*02, DQB1*0301, or DQB1*0602); Mild risk: HLA-DQA1*05-DQB1*02/y (y not DQA1*0201-DQB1*02, DQB1*0301, DQB1*0602, or DQB1*0603) and HLA-DQA1*03-DQB1*02/y (y not DQA1*0201-DQB1*02, DQB1*0301, DQB1*0602, or DQB1*0603).

### Differences in Correlation Coefficients Between Fatty Acids and Immunological Markers in Cases Compared to Control Children

Altogether 14 statistically significant (*p* < 0.01) correlations between fatty acids and immunological markers were observed in cord serum in case children and 22 in control children. At 6 months of age, there were 39 significant correlations in cases and 37 in controls, and at 12 months of age, 11 in cases and 10 in controls (**Figure 1**). In cord serum samples, 14 correlations were statistically different in strength (*p* < 0.05) between case and control children (**Table 2**), 24 at the age of 6 months (**Table 3**), and 10 at the age of 12 months (**Table 4**).

**Table 2:** Differences in Spearman correlation coefficients between cord serum fatty acids and immunological markers by case–control status.

Fatty acid and immunological marker, between which correlation was tested		Correlation coefficient in cases (*n* = 63)		Correlation coefficient in controls (*n* = 100)		Difference between cases and controls
Fatty acid	Immunological marker	*r*	*p*-value	*r*	*p*-value	Adjusted *p*-value
Myristic 14:0	IL-6	0.431	**<0.001**	0.031	0.763	**0.019**
Pentadecanoid 15:0	CXCL10	−0.331	**0.008**	0.104	0.305	**0.019**
Iso-heptadecanoid ai17:0	CXCL10	−0.345	**0.006**	0.169	0.093	**0.019**
Stearic 18:0	IL-6	−0.374	**0.003**	0.017	0.865	**0.022**
Eicosanoid 20:0	IL-6	−0.350	**0.005**	0.079	0.433	**0.019**
Eicosanoid 20:0	IL-17	−0.332	**0.008**	0.087	0.387	**0.019**
Tetracosanic 24:0	IL-17	−0.391	**0.002**	0.018	0.860	**0.019**
Tetracosanic 24:0	FGF-2	−0.359	**0.004**	0.119	0.240	**0.019**
Vaccenic 18:1n-7	TNF-β	0.106	0.410	−0.279	**0.005**	**0.024**
Oleic 18:1n-9	GM-CSF	0.151	0.236	−0.258	**0.010**	**0.021**
Oleic 18:1n-9	IFN-α2	0.103	0.421	−0.316	**0.001**	**0.019**
Oleic 18:1n-9	IL-13	0.070	0.585	−0.296	**0.003**	**0.029**
DPA 22:5n-3	IL-13	−0.059	0.648	0.282	**0.004**	**0.039**
DPA 22:5n-3	IL-1α	−0.078	0.543	0.307	**0.002**	**0.024**

The table includes all the correlation coefficients that were significant for cases or controls (*p* < 0.01) and different between them using Fisher’s *r* to *z*-transformation for Spearman correlation and *z*-test (*p* < 0.05) and p-values were further adjusted with the Benjamini–Hochberg method. Significant p-values are bolded.

**Table 3:** Differences in Spearman correlation coefficients between serum fatty acids and immunological markers at 6 months of age by case–control status.

Fatty acid and immunological marker, between which correlation was tested		Correlation coefficients in cases (*n* = 69)		Correlation coefficients in controls (*n* = 111)		Difference between cases and controls
Fatty acid	Immunological marker	*r*	*p*-value	*r*	*p*-value	Adjusted *p*-value
Pentadecanoid 15:0	IL-9	−0.396	**0.001**	−0.067	0.483	**0.041**
Palmitic 16:0	G-CSF	−0.112	0.358	0.248	**0.009**	**0.041**
Heptadecanoid i17:0	TGF-α	−0.324	**0.007**	0.005	0.961	**0.041**
Iso-heptadecanoid ai17:0	IL-9	−0.391	**0.001**	−0.070	0.466	**0.041**
Stearic 18:0	GM-CSF	−0.313	**0.009**	0.037	0.703	**0.041**
Eicosanoid 20:0	TGF-α	0.001	0.993	0.309	**0.001**	**0.048**
Eicosanoid 20:0	IL-1rα	−0.070	0.566	0.307	**0.001**	**0.041**
Eicosanoid 20:0	IL-5	−0.060	0.624	0.261	**0.006**	**0.043**
Vaccenic 18:1n-7	IL-9	−0.440	<0.001	−0.086	0.367	**0.041**
LA 18:2n-6	IL-9	0.365	**0.002**	0.053	0.580	**0.043**
LA 18:2n-6	MCP-3	0.371	**0.002**	0.044	0.650	**0.041**
LA 18:2n-6	IL-1rα	0.360	**0.002**	0.025	0.792	**0.041**
LA 18:2n-6	IL-6	0.426	**<0.001**	0.030	0.753	**0.041**
LA 18:2n-6	TNF-β	0.354	**0.003**	0.041	0.671	**0.043**
DGLA 20:3n-6	IL-10	0.097	0.427	−0.263	**0.005**	**0.041**
DGLA 20:3n-6	TGF-α	0.014	0.906	−0.336	**<0.001**	**0.041**
DGLA 20:3n-6	IL-1rα	0.126	0.303	−0.246	**0.009**	**0.041**
AA 20:4n-6	CXCL10	0.324	**0.007**	−0.038	0.691	**0.041**
Adrenic 22:4n-6	G-CSF	0.055	0.656	−0.302	**0.001**	**0.041**
Adrenic 22:4n-6	IL-15	0.127	0.299	−0.257	**0.007**	**0.041**
Adrenic 22:4n-6	IL-6	0.145	0.235	−0.256	**0.007**	**0.041**
CLA 18:2n-7	IL-9	−0.411	**0.001**	−0.037	0.703	**0.041**
DMA18	GM-CSF	−0.394	**0.001**	−0.061	0.522	**0.041**
DMA18	IL-9	−0.401	**0.001**	−0.065	0.497	**0.041**
Ratio of n-6:n-3	TGF-α	0.424	**0.000**	0.101	0.290	**0.041**

The table includes all the correlation coefficients that were significant for cases or controls (*p* < 0.01) and different between them using Fisher’s *r* to *z*-transformation for Spearman correlation and *z*-test (*p* < 0.05) and *p*-values were further adjusted with the Benjamini–Hochberg method. Significant *p*-values and bolded.

**Table 4:** Differences in Spearman correlation coefficients between serum fatty acids and immunological markers at 12 months of age by case–control status.

Fatty acid and immunological marker, between which correlation was tested		Correlation coefficients in cases (*n* = 63)		Correlation coefficients in controls (*n* = 102)		Difference between cases and controls
Fatty acid	Immunological marker	*r*	*p*-value	*r*	*p*-value	Adjusted *p*-value
Palmitic 16:0	IL-9	−0.340	**0.007**	−0.021	0.836	**0.048**
Docosanoid 22:0	IL-17	0.351	**0.005**	0.000	0.997	**0.042**
Tetracosanic 24:0	IL-17	0.339	**0.007**	−0.105	0.295	**0.016**
Tetracosanic 24:0	IL-1ra	0.348	**0.005**	−0.115	0.250	**0.015**
Tetracosanic 24:0	IL-1α	0.214	0.093	−0.259	**0.009**	**0.015**
Tetracosanic 24:0	IL-9	0.335	**0.007**	−0.180	0.071	**0.015**
Oleic 18:1n-9	IFN-γ	−0.324	**0.010**	0.100	0.319	**0.019**
Palmitoleic 16:1n-9	CXCL1	−0.334	**0.007**	0.000	1.000	**0.046**
Eicosenoic 20:1n-9	CXCL1	−0.369	**0.003**	0.034	0.738	**0.021**
LA 18:2n-6	IL-17	0.077	0.549	−0.261	**0.008**	**0.046**

The table includes all the correlation coefficients that were significant for cases or controls (*p* < 0.01) and different between them using Fisher’s *r* to *z*-transformation for Spearman correlation and *z*-test (*p* < 0.05) and *p*-values were further adjusted with the Benjamini–Hochberg method. Significant *p*-values were bolded.

### Correlations Between Cord Serum Fatty Acids and Immunological Markers

In case children’s cord serum, SFAs correlated with several immunological markers. Among the SFAs, 14:0 correlated directly with IL-6, 15:0 and ai17:0 correlated inversely with CXCL10, 18:0 and 20:0 correlated inversely with IL-6, and 24:0 correlated inversely with FGF-2 and IL-17 (**Table 2**).

In control children’s cord serum, n-3 PUFAs and MUFAs correlated with a series of immunological markers, while these associations were not seen in case children (**Table 2** and **Figure 1**). In control children, DPA correlated directly with IL-1α and IL-13; oleic acid correlated inversely with GM-CSF, IFN-α2, and IL-13; and vaccenic acid correlated inversely with TNF-β (**Table 2**).

### Correlations Between Serum Fatty Acids and Immunological Markers at 6 Months of Age

In case children at 6 months of age, several SFAs correlated inversely with certain immunological markers: 15:0 with IL-9, a17:0 with TGF-α, ai17:0 with IL-9, and 18:0 with GM-CSF. Vaccenic acid correlated inversely with IL-9; LA correlated directly with IL-6, IL-9, MCP-3, IL-1ra, and TNF-β; and AA correlated directly with CXCL10 in case children. In addition, CLA and DMA18 correlated inversely with IL-9, and DMA18 correlated inversely with GM-CSF. These associations were not seen in control children (**Table 3**).

In control children at 6 months of age, 16:0 correlated directly with G-CSF and 20:0 correlated directly with TGF-a, IL-1ra, and IL-5. Of n-6 PUFAs, DGLA correlated inversely with IL-10, TGF-α, and IL1ra, and adrenic acid correlated inversely with G-CSF, IL-6, and IL-15. These associations were not observed in case children (**Table 3**).

### Correlations Between Serum Fatty Acids and Immunological Markers at 12 Months of Age

In case children at 12 months of age, 16:0 correlated inversely with IL-9, 22:0 and 24:0 correlated directly with IL-17, and 24:0 correlated directly with IL-9, IL-1ra, and IL-1α. Oleic acid correlated inversely with IFN-γ and palmitoleic and eicosenoic acid with CXCL1 in cases. In contrast to cases, there was an inverse correlation between LA and IL-17. In controls, 24:0 correlated inversely with IL-1α (**Table 4**).

Immunological markers that showed a statistically significant association with fatty acids did not differ by breastfeeding status or maternal type 1 diabetes. Furthermore, weight z score did not correlate with fatty acids or immunological markers in a way that it would affect the interpretation of the results.

## Discussion

We observed differences in the associations between serum fatty acids and immunological markers during the first year of life in case compared to control children. In general, in children who developed islet autoimmunity, SFAs showed more associations with immunological markers, while in control children, PUFAs showed stronger associations. These observations are in line with the findings of several large-scale birth cohort studies that high levels of n-3 PUFAs were associated with decreased risk of islet autoimmunity (9, 10, 31), while SFAs tended to be associated with increased risk (29–31).

It was previously observed in the TRIGR study that several SFAs (15:0, i17:0 and ai17:0, 18:0) and CLA were associated with increased risk of islet autoimmunity (30). We observed that in case children, several SFAs and CLA were associated with many immunological markers [CXCL10 (IP-10), IL-6, IL-9, IL-17, and CM-CSF] that have been linked to the development of type 1 diabetes (3–6, 8, 39). In our study, associations between most SFAs (15:0, ai17:0, 18:0, 20:0, and 24:0) and these cytokines were inverse in cord serum and at 6 months of age, but at the age of 12 months, associations between 22:0 and 24:0 with IL-17 were direct. During the first year of life, the immune system is developing rapidly, and also the dietary intake of fatty acid changes due to increasing food variety in the child’s diet, and thus associations between circulating fatty acids and immunological markers may vary by age. Our results indicate that changes in fatty acid intake and/or metabolism occur in case children, detected as increased SFA and CLA levels, concurrently with alterations in patterns of immunological markers. Whether the observed correlations in our study between SFAs and inflammatory immunological markers indicate causality is unknown. It has been shown that SFAs have direct immunomodulatory effects. They have been shown to stimulate proliferation of T cells and their differentiation to proinflammatory phenotypes Th1 and Th17 (32), which is in line with our finding at 12 months of age. On the other hand, CLA has shown capacity to downregulate pro-inflammatory cytokines, such as IL-6, TNFα, IFNγ, and IL-1β in cell culture studies (40), which is in accordance with our result that CLA was inversely associated with IL-9.

In case children, the observed inverse relations at birth and at 6 months of age between SFAs and CLA linked to islet autoimmunity and several proinflammatory immunological markers linked to type 1 diabetes development may be explained by the fact that higher levels of proinflammatory immunological markers during the first months of life may be actually beneficial, while at later age, they may indicate inflammation. At birth, the infant’s immunity is in a tolerogenic state in order to tolerate maternal antigens and stress (innate immunity and Th1 responses are suppressed). After birth, dynamic co-development between the innate and adaptive immune system as well as microbes, are needed for developing regulated immune responses against antigens and pathogens. Cytokines mediate the activation of these early innate immune responses and the training of the adaptive immune system (12).

In control children, PUFAs were more strongly associated with many more immunological markers compared to case children. Cord serum DPA was directly associated with IL-13 and IL-1α in control children. IL-13 is secreted by activated Th2 cells and it has been associated with downregulation of several macrophage and monocyte-derived pro-inflammatory cytokines (41). In the TRIGR study, it was observed that cord serum DPA (n-3 PUFA) tended to be associated with decreased risk of later islet autoimmunity (30). Three other large-scale birth cohort studies (DIPP, DAISY, and TEDDY) have showed that n-3 PUFA (ALA, EPA, DPA, or DHA) intake or their increased proportions in serum or erythrocytes were associated with a reduced risk of islet autoimmunity (9, 10, 31). In animal models and clinical trials, an n-3 PUFA-rich diet has been shown to decrease the production of inflammatory cytokines and increase the secretion of anti-inflammatory cytokines, increase regulatory T cells, and reduce T-cell proliferation, activation, and differentiation into proinflammatory Th1 and Th17 cells (32). In addition to modulating immune responses and inflammatory mediators, n-3 PUFAs could also modulate the diversity and abundance of the gut microbiota, particularly the ratio of *Firmicutes* and *Bacteroides* (42). Changes in gut microbiota such as decreased relative abundance in *Firmicutes* and increased relative proportion of *Bacteroides* have been observed already at early age in children who later on develop type 1 diabetes (43). Furthermore, n-3 PUFA may affect gut microbiota also by regulating the levels of short-chain fatty acids or short-chain fatty acid salts (42). On the other hand, gut microbiota could modulate the absorption and metabolism of n-3 PUFAs and thus its intake and function (42).

Associations between n-6 PUFA and immunological markers were heterogenous. In control children, certain n-6 PUFAs (DGLA and adrenic acid) correlated inversely with several cytokines (IL-10, TGF-α, IL-1ra, G-CSF, IL-15, and IL-6) at 6 months of age. At 12 months of age, control children showed an inverse association between LA and IL-17, out of which the latter has been linked to type 1 diabetes (15, 19, 21). In turn, in case children at the age of 6 months, n-6 LA and AA were directly associated with cytokines (IL-6, IL-9, and CXCL10), which have been previously linked to pre-clinical and clinical type 1 diabetes (3–6, 39). Altogether, our findings indicate that both n-3 and n-6 PUFAs, particularly in the early months, may be associated with the pattern of circulating cytokines, and thus possibly play a role in the maturation of the immune system and longer-term immune responses.

In our study, DMA18 also correlated inversely in case children at 6 months of age with IL-9 and GM-CSF, i.e., cytokines that have been linked to type 1 diabetes (8, 21, 39). DMA18 is a plasmalogen, which is an unusual subgroup of phospholipids in membranes. Plasmalogens have been implicated to play an important role in many cell functions, such as cell signaling and protection against reactive oxygen species. The metabolism of plasmalogens is related to both n-3 and n-6 PUFAs: plasmalogens may serve as reservoir for PUFAs, and thus, plasmalogen deficiency may affect PUFA levels, and on the other hand, the presence of PUFA is needed for the biological activity of plasmalogens (44, 45). One of the plasmalogen species is choline plasmalogen (46). Interestingly, cord blood choline-containing phospholipids (47) and unsaturated phosphatidylcholines in infancy (27) were found to be low in children who developed islet autoimmunity.

There were no clear patterns for associations between MUFAs and immunological markers. In cord serum from control children, some MUFAs showed inverse correlation: vaccenic acid with TNF-β and oleic acid with GM-CSF, IFN-α2, and IL-13. In cases at 6 months of age, vaccenic acid correlated inversely with IL-9, and further at 12 months of age, oleic acid was inversely related to IFN-γ and palmitoleic and eicosenoic acids were inversely related to CXCL1. As far as we know, there are no earlier evidence of these kinds of associations.

The strengths of the present study include a unique sample set from several different countries, and the possibility to use unthawed samples for immunological marker analysis. We analyzed a relatively large number of fatty acids and immunological markers, which enables a wider view of complex immunomodulatory networks. A limitation is that our study design does not allow us to draw conclusions about causality regarding associations between fatty acids and immunological markers, and the results might reflect some other factors related to disease etiology. A limitation of the study is the unavailability of genetic factors related to the regulation of immunological markers (48) and fatty acid metabolism, such as polymorphism in genes encoding fatty acid desaturases and elongases (14). Generalizability of our results to the whole population remains open since our study population comprised high-risk children due to their HLA-conferred risk of type 1 diabetes and having a first-degree relative/s with type 1 diabetes.

### Conclusions

The present findings provide mechanistic suggestions to the previously found associations between fatty acids and the risk of type 1 diabetes. Our study showed that correlation patterns between circulating fatty acids and immunological markers were distinctly different among those children who developed islet autoimmunity compared to control children already at birth continuing across the first year of life. In case children, associations between SFAs and immunological markers were observed for several markers linked to type 1 diabetes development, and some of these SFAs were associated with the development of islet autoimmunity in the current study as well. The results support the view that autoimmunity may be a later stage consequence of metabolic and immunologic disturbances occurring in early infancy. Therefore, the critical time window for preventive interventions may be during the fetal development or in the early postnatal period. Findings indicate that fatty acids could have immunomodulatory potential in the early phase of the disease development, already prior to the appearance of the first signs of islet autoimmunity. In our analyses, the nodes showing the most correlative associations with each other may provide elements for creating new combinatorial factors for better understanding of the complex interplay between factors linked to the pathogenesis of type 1 diabetes. Further research is required to understand the role of intake of fatty acids and fatty acid metabolism and their effect on immune responses in the development of type 1 diabetes.

## Data Availability Statement

The raw data supporting the conclusions of this article will be made available by the authors, without undue reservation.

## Ethics Statement

The studies involving human participants were reviewed and approved by the ethics committees of all participating centers. The study was conducted according to the standards of the Declaration of Helsinki. Written informed consent to participate in this study was provided by the participants’ legal guardian/next of kin.

## Author Contributions

SN, MM, and SV were responsible for conception and design of the study. MK, JK, SV, and OV were responsible for the acquisition of data. JH, AV, TH, MK, IE, and PA were responsible for laboratory analysis. DC and RA did statistical analysis. SN, MM, SV, and LH drafted the article with contributions from JH and MK. MK and SV are the guarantors of this work. All authors contributed to the interpretation of the data and critically reviewed and approved the version to be published.

## Funding

This work was supported by National Institutes of Health (grants 1DP3DK106918-01, HD040364, HD042444, and HD051997), the Eunice Kennedy Shriver National Institute of Child Health and Development (NICHD), National Institute of Diabetes and Digestive and Kidney Diseases, Canadian Institutes of Health Research, JDRF, the Commission of the European Communities (specific RTD programme Quality of Life and Management of Living Resources, contract QLK1-2002-00372 Diabetes Prevention), the European Foundation for the Study of Diabetes/JDRF/Novo Nordisk Focused Research Grant, Academy of Finland (Centre of Excellence in Molecular Systems Immunology and Physiology Research 2012-2017, Decision No. 250114), Dutch Diabetes Research Foundation, and Finnish Diabetes Research Foundation. The study sponsors were not involved in the design of the study; the collection, analysis, and interpretation of data; writing the report; or the decision to submit the report for publication.

## Conflict of Interest

The authors declare that the research was conducted in the absence of any commercial or financial relationships that could be construed as a potential conflict of interest.

## Publisher’s Note

All claims expressed in this article are solely those of the authors and do not necessarily represent those of their affiliated organizations, or those of the publisher, the editors and the reviewers. Any product that may be evaluated in this article, or claim that may be made by its manufacturer, is not guaranteed or endorsed by the publisher.

## Abbreviations

AA, arachidonic acid; ALA, alphalinolenic acid; CLA, conjugated linoleic acid; DGLA, dihomogammalinolenic acid; DPA, docosapentaenoic acid; DHA, docosahexaenoic acid; DMA, dimethylacetal; EPA, eicosapentaenoic acid; GADA, glutamic acid decarboxylase autoantibody; HLA, human leukocyte antigen; IAA, insulin autoantibody; IA-2A, insulinoma-associated antigen-2 autoantibody; ICA, islet cell autoantibody; LA, linoleic acid; MUFA, monounsaturated fatty acid; PUFA, polyunsaturated fatty acid; SFA, saturated fatty acid; ZnT8A, zinc transporter-8 autoantibody.
