# Supplementary material for: Associations Between Serum Fatty Acids and Immunological Markers in Children Developing Islet Autoimmunity—The TRIGR Nested Case–Control Study
Source: Front Immunol. 2022 May 25;13:858875. doi: 10.3389/fimmu.2022.858875 (PMC9175567; doi:10.3389/fimmu.2022.858875)
Supplement: Supplementary file 1 [file DataSheet_1.pdf]

Supplementary file for manuscript Niinistö et al. Associations between serum fatty acids and immunological markers in children developing islet autoimmunity – the TRIGR nested case-control study

Supplementary Table 1. Distribution of serum proportion of fatty acids (median, IQR)

	0 months		6 months		12 months	
**Fatty acid (% of total)**	Cases n=63	Controls n=100	Cases n=69	Controls n=111	Cases n=63	Controls n=102
SFAs						
14:0	0.85 (0.68, 0.97)	0.82 (0.70, 0.97)	1.58 (1.10, 2.03)	1.36 (1.02, 1.77)	1.26 (0.92, 1.92)	1.30 (1.00, 1.87)
15:0	0.20 (0.18, 0.25)	0.19 (0.18, 0.23)	0.19 (0.11, 0.23)	0.15 (0.08, 0.20)	0.21 (0.15, 0.28)	0.20 (0.15, 0.29)
16:0	25.55 (24.81, 26.62)	25.73 (24.95, 26.61)	22.45 (21.52, 23.87)	22.12 (21.25, 23.14)	22.77 (21.38, 23.75)	22.56 (21.24, 24.19)
i17:0	0.15 (0.13, 0.18)	0.14 (0.12, 0.16)	0.09 (0.05, 0.11)	0.07 (0.03, 0.10)	0.11 (0.06, 0.16)	0.11 (0.06, 0.15)
ai17:0	0.15 (0.12, 0.17)	0.14 (0.12, 0.17)	0.10 (0.05, 0.14)	0.09 (0.04, 0.12)	0.12 (0.08, 0.16)	0.12 (0.08, 0.16)
18:0	9.58 (8.90, 10.25)	9.79 (9.34, 10.24)	7.93 (7.34, 8.98)	8.06 (7.32, 8.68)	7.68 (7.03, 8.37)	7.34 (6.80, 7.91)
20:0	0.31 (0.26, 0.35)	0.31 (0.27, 0.34)	0.21 (0.20, 0.23)	0.21 (0.19, 0.23)	0.19 (0.17, 0.22)	0.19 (0.17, 0.20)
22:0	0.42 (0.35, 0.46)	0.45 (0.39, 0.50)	0.31 (0.26, 0.41)	0.34 (0.29, 0.42)	0.34 (0.26, 0.40)	0.32 (0.27, 0.38)
24:0	0.42 (0.35, 0.47)	0.46 (0.38, 0.50)	0.26 (0.22, 0.33)	0.29 (0.24, 0.34)	0.28 (0.24, 0.33)	0.28 (0.24, 0.32)
MUFAs						
16:1n-7 (PTOL)	3.91 (3.51, 4.73)	4.17 (3.62, 4.72)	1.54 (1.22, 1.78)	1.39 (1.18, 1.69)	1.61 (1.21, 1.92)	1.51 (1.23, 2.04)
16:1n-9 (PTOL2)	0.83 (0.74, 0.95)	0.79 (0.72, 0.90)	0.33 (0.30, 0.37)	0.32 (0.29, 0.36)	0.34 (0.31, 0.40)	0.33 (0.29, 0.38)
18:1n-7 (cVAKS)	3.60 (3.28, 3.95)	3.50 (3.29, 3.85)	2.21 (1.56, 2.40)	2.04 (1.58, 2.30)	2.05 (1.84, 2.29)	1.99 (1.81, 2.26)
18:1n-9 (OLE)	19.67 (18.32, 21.62)	19.20 (18.15, 20.21)	26.53 (23.55, 28.89)	25.79 (23.28, 27.85)	25.81 (22.82, 27.68)	26.03 (22.97, 28.67)
24:1n-9 (NERVO)	0.65 (0.55, 0.77)	0.70 (0.60, 0.81)	0.50 (0.39, 0.58)	0.52 (0.44, 0.63)	0.53 (0.44, 0.64)	0.53 (0.43, 0.65)
20:1n-9 (EIKE)	0.15 (0.12, 0.18)	0.15 (0.13, 0.17)	0.26 (0.23, 0.31)	0.25 (0.23, 0.27)	0.26 (0.21, 0.32)	0.27 (0.22, 0.31)
n-6 PUFA	27.09 (25.04, 29.69)	27.34 (25.82, 29.29)	32.13 (27.94, 35.20)	33.07 (30.64, 36.09)	33.06 (29.66, 36.23)	33.37 (28.63, 36.54)
18:2n-6 (LA)	11.14 (9.91, 12.15)	11.05 (10.03, 12.93)	26.42 (23.19, 28.75)	27.56 (25.08, 29.90)	27.30 (25.15, 29.76)	27.51 (24.13, 30.13)
203n-6 (DGLA)	3.17 (2.80, 3.56)	3.20 (2.79, 3.60)	1.00 (0.81, 1.17)	1.00 (0.83, 1.24)	1.03 (0.86, 1.25)	1.01 (0.84, 1.21)
20:4n-6 (ARA)	12.50 (10.60, 13.89)	12.27 (11.14, 13.72)	4.26 (3.31, 5.71)	4.52 (3.37, 5.80)	4.26 (3.37, 5.16)	3.94 (2.92, 5.52)
22:4n-6 (DTETR)	0.55 (0.46, 0.63)	0.52 (0.46, 0.61)	0.17 (0.14, 0.23)	0.18 (0.15, 0.21)	0.19 (0.15, 0.22)	0.19 (0.14, 0.23)
n-3 PUFA	4.50 (3.71, 5.36)	4.40 (3.69, 5.33)	2.52 (1.88, 3.44)	2.34 (1.63, 3.63)	2.43 (1.91, 3.00)	2.44 (1.87, 3.19)
18:3n-3 (ALA)	0.20 (0.16, 0.29)	0.18 (0.13, 0.26)	0.66 (0.51, 0.80)	0.60 (0.47, 0.74)	0.67 (0.49, 0.80)	0.65 (0.51, 0.90)
20:5n-3 (EPA)	0.28 (0.19, 0.38)	0.29 (0.20, 0.41)	0.21 (0.13, 0.34)	0.18 (0.11, 0.37)	0.25 (0.16, 0.39)	0.25 (0.17, 0.36)
22:5n-3 (DPA)	0.29 (0.20, 0.39)	0.29 (0.20, 0.38)	0.33 (0.24, 0.48)	0.33 (0.23, 0.48)	0.34 (0.27, 0.43)	0.34 (0.24, 0.45)
22:6n-3 (DHA)	3.79 (2.98, 4.49)	3.63 (3.05, 4.36)	1.30 (0.86, 1.89)	1.27 (0.70, 2.04)	1.03 (0.76, 1.57)	1.11 (0.73, 1.54)
18:2n-7 (CLA)	0.27 (0.23, 0.31)	0.24 (0.20, 0.30)	0.20 (0.10, 0.24)	0.15 (0.07, 0.20)	0.20 (0.13, 0.27)	0.19 (0.12, 0.30)
DMA16	0.47 (0.38, 0.55)	0.50 (0.42, 0.55)	0.37 (0.31, 0.43)	0.39 (0.32, 0.44)	0.37 (0.29, 0.46)	0.37 (0.31, 0.46)
DMA18	0.15 (0.12, 0.17)	0.15 (0.13, 0.17)	0.24 (0.17, 0.31)	0.24 (0.17, 0.33)	0.24 (0.17, 0.30)	0.23 (0.18, 0.29)

Supplementary Table 2. Distribution of serum immunological markers (median, IQR).

	0 months		6 months		12 months	
**Immunological marker (pg/ml)**	Cases n=63	Controls n=100	Cases n=69	Controls n=111	Cases n=63	Controls n=102
**Chemokine**						
CCL2	841.7 (663.5, 1113)	849.6 (627.9, 1112)	855.1 (636.8, 1076)	872.3 (715.3, 1141)	748.1 (550.0, 975.1)	791.9 (632.6, 1012)
CCL3	13.7 (9.5, 43.1)	16.8 (9.2, 39.1)	11.3 (7.6, 20.8)	11.8 (8.0, 19.3)	11.6 (6.9, 24.7)	10.8 (7.4, 23.7)
CCL4	113.4 (79.3, 153.6)	111.6 (86.2, 156.0)	111.1 (72.9, 141.7)	107.3 (83.8, 139.2)	79.3 (63.4, 131.6)	86.0 (58.3, 121.7)
CCL7	3.2 (3.2, 31.1)	3.2 (3.2, 38.1)	3.2 (3.2, 19.1)	3.2 (3.2, 35.2)	45.9 (3.2, 190.9)	40.1 (3.2, 131.7)
CCL11	136.4 (97.2, 192.0)	122.3 (97.6, 190.5)	138.4 (97.3, 199.0)	157.1 (123.3, 201.4)	147.2 (93.8, 217.5)	174.9 (127.9, 233.3)
CCL22	1614 (1353, 2067)	1801 (1371, 2207)	2293 (1887, 2940)	2787 (2167, 3217)	1768 (1463, 2367)	1971 (1474, 2492)
CXCL1	1556 (909, 2149)	1738 (1188, 2494)	2421 (1726, 3101)	2488 (1835, 4388)	2280 (1712, 3169)	2640 (1884, 4133)
CXCL10	171.0 (114.9, 259.2)	161.3 (113.4, 217.0)	204.1 (136.2, 354.8)	224.6 (169.1, 391.7)	276.3 (187.0, 463.0)	279.2 (190.9, 391.1)
CXC3CL1	77.9 (25.3, 134.1)	75.6 (41.1, 122.3)	73.2 (41.4, 127.5)	83.7 (49.0, 139.5)	77.9 (32.1, 138.1)	81.0 (44.7, 132.1)
**Growth factor**						
EGF	587.0 (313.2, 990.2)	750.0 (341.6, 1204.6)	246.0 (97.0, 727.9)	329.3 (168.0, 691.8)	201.0 (88.5, 542.5)	253.7 (89.3, 464.0)
FGF-2	99.3 (59.5, 205.0)	135.6 (57.1, 257.8)	80.4 (64.6, 99.1)	76.6 (61.3, 98.6)	91.8 (71.3, 126.1)	87.0 (65.7, 115.2)
VEGF	457.9 (228.4, 898.0)	503.6 (258.9, 881.2)	288.4 (186.1, 417.6)	222.5 (130.5, 420.8)	251.6 (140.6, 461.0)	224.8 (132.3, 344.9)
**Innate immune cytocine**						
IFNα2	16.8 (2.4, 67.8)	21.8 (2.4, 65.0)	26.7 (2.4, 59.8)	41.6 (11.3, 72.2)	37.9 (16.6, 87.9)	43.6 (20.2, 82.0)
IL-1ra	129.1 (37.2, 497.5)	100.5 (35.9, 269.0)	16.2 (8.6, 58.7)	16.4 (8.6, 90.9)	81.6 (16.2, 188.2)	62.7 (17.0, 237.2)
IL-1α	6.3 (6.3, 62.4)	9.1 (6.3, 54.6)	6.3 (6.3, 6.3)	6.3 (6.3, 11.9)	6.3 (6.3, 34.5)	6.3 (6.3, 50.5)
IL-1β	0.5 (0.5, 6.5)	0.5 (0.5, 3.6)	0.8 (0.5, 2.3)	0.9 (0.5, 2.6)	1.4 (0.8, 4.7)	1.6 (0.7, 3.8)
IL-6	4.2 (0.7, 18.6)	3.3 (0.7, 37.4)	0.7 (0.7, 10.1)	0.7 (0.7, 12.9)	9.0 (0.7, 58.4)	8.0 (0.7, 31.9)
IL-12p40	6.4 (6.4, 14.7)	6.4 (6.4, 18.4)	6.4 (6.4, 17.1)	6.4 (6.4, 18.0)	21.9 (6.4, 52.0)	16.4 (6.4, 39.9)
IL-12p70	0.5 (0.5, 0.7)	0.5 (0.5, 2.0)	1.2 (0.5, 4.6)	1.3 (0.5, 4.5)	2.3 (0.5, 6.2)	1.9 (0.5, 7.1)
**Other cytokine**						
IL-2	0.8 (0.8, 0.9)	0.8 (0.8, 0.8)	1.2 (0.8, 2.2)	1.0 (0.8, 2.1)	1.6 (0.9, 3.0)	1.6 (0.8, 2.9)
IL-3	0.5 (0.5, 0.5)	0.5 (0.5, 0.5)	0.5 (0.5, 0.5)	0.5 (0.5, 0.5)	0.5 (0.5, 1.0)	0.5 (0.5, 1.2)
IL-7	1.2 (1.2, 6.2)	2.6 (1.2, 7.1)	4.5 (1.2, 10.4)	5.0 (1.2, 15.4)	6.6 (2.8, 13.5)	8.4 (2.8, 17.4)
IL-8	21.4 (12.3, 325.7)	33.1 (13.5, 188.6)	15.3 (10.8, 36.9)	16.8 (10.3, 35.2)	17.9 (12.6, 51.9)	18.4 (10.7, 51.5)
IL-9	1.0 (1.00, 3.6)	1.7 (1.0, 4.5)	1.0 (1.0, 3.2)	1.0 (1.0, 3.7)	1.1 (1.0, 5.4)	1.6 (1.0, 6.7)
IL-15	0.9 (0.9, 3.6)	0.9 (0.9, 3.5)	0.9 (0.9, 1.4)	0.9 (0.9, 2.0)	0.9 (0.9, 3.4)	1.0 (0.9, 3.5)
**Other inflammation related markers**						
FIt-3L	7.4 (3.5, 29.0)	9.6 (3.5, 30.1)	3.5 (3.5, 3.5)	3.5 (3.5, 3.5)	3.5 (3.5, 3.5)	3.5 (3.5, 3.5)
G-CSF	67.4 (42.9, 124.3)	76.9 (35.4, 108.7)	33.8 (10.8, 64.5)	42.9 (22.2, 75.9)	49.3 (20.9, 86.8)	49.5 (21.6, 97.2)
GM-CSF	7.5 (7.5, 11.3)	7.5 (7.5, 12.3)	15.6 (10.0, 24.5)	16.7 (11.2, 24.5)	16.9 (12.1, 25.6)	17.7 (11.6, 27.7)
sCD40L	5.0 (5.0, 3491.1)	5.0 (5.0, 1937.8)	3182 (556, 5796)	3558 (381, 6943)	3743 (1481, 6311)	4783 (2325, 6861)
TGF-α	12.8 (6.9, 29.1)	15.8 (7.7, 31.6)	3.8 (2.2, 7.9)	3.8 (1.8, 8.1)	5.9 (3.2, 10.9)	4.9 (2.7, 8.4)
TNF-β	1.0 (1.0, 1.0)	1.0 (1.0, 1.0)	1.0 (1.0, 1.0)	1.0 (1.0, 3.4)	11.9 (1.0, 104.3)	5.4 (1.0, 65.2)

**Th1 cytokine**						
TNF-α	53.2 (43.3, 73.5)	57.9 (46.6, 72.7)	54.6 (46.1, 71.3)	60.0 (46.6, 73.3)	55.0 (40.5, 72.4)	51.8 (44.4, 66.6)
IFN-γ	1.7 (0.6, 6.8)	1.8 (0.6, 4.8)	7.8 (5.6, 15.8)	8.8 (5.6, 14.4)	14.4 (6.3, 22.3)	11.1 (6.5, 24.7)
**Th17 cytokine**						
IL-17	0.6 (0.6, 1.2)	0.6 (0.6, 1.5)	3.9 (2.4, 5.1)	3.9 (2.6, 7.4)	4.2 (2.6, 9.5)	4.6 (2.6, 8.8)
**Th2 cytokine**						
IL-4	31.0 (9.1, 60.7)	33.5 (8.7, 64.8)	67.2 (30.2, 126.3)	67.7 (35.5, 115.2)	70.7 (43.0, 129.9)	81.7 (44.8, 132.7)
IL-5	0.7 (0.4, 1.6)	0.5 (0.4, 1.5)	0.7 (0.4, 1.4)	0.9 (0.4, 2.8)	1.5 (0.4, 5.6)	1.6 (0.5, 5.1)
IL-13	1.0 (1.0, 5.2)	1.0 (1.0, 6.5)	1.0 (1.0, 1.5)	1.0 (1.0, 2.6)	8.2 (1.0, 45.2)	6.7 (1.0, 47.9)
**Treg cytokine**						
IL-10	6.6 (3.1, 16.5)	10.4 (3.8, 18.1)	6.2 (3.4, 13.5)	10.1 (4.8, 17.7)	12.7 (8.9, 20.0)	11.3 (5.6, 22.7)
